# Key Genes Associated With Non-Alcoholic Fatty Liver Disease and Polycystic Ovary Syndrome

**DOI:** 10.3389/fmolb.2022.888194

**Published:** 2022-05-25

**Authors:** Yong Chen, Leikai Ma, Zhouling Ge, Yizhao Pan, Lubin Xie

**Affiliations:** ^1^ Department of Anesthesiology, The First Affiliated Hospital of Wenzhou Medical University, Wenzhou, China; ^2^ Department of Obstetrics and Gynecology, The First Affiliated Hospital of Wenzhou Medical University, Wenzhou, China; ^3^ Department of Respiratory Medicine, The Third Affiliated Hospital of Shanghai University (Wenzhou People’s Hospital), Wenzhou, China

**Keywords:** PCOS (polycystic ovarian syndrome, NAFLD (non alcoholic fatty liver disease), bioinformatics analysis, differentially expressed genes (DEG’s), MiRNA-mRNA regulatory network

## Abstract

**Background:** Polycystic ovary syndrome (PCOS) is the most common metabolic and endocrinopathies disorder in women of reproductive age and non-alcoholic fatty liver (NAFLD) is one of the most common liver diseases worldwide. Previous research has indicated potential associations between PCOS and NAFLD, but the underlying pathophysiology is still not clear. The present study aims to identify the differentially expressed genes (DEGs) between PCOS and NAFLD through the bioinformatics method, and explore the associated molecular mechanisms.

**Methods:** The microarray datasets GSE34526 and GSE63067 were downloaded from Gene Expression Omnibus (GEO) database and analyzed to obtain the DEGs between PCOS and NAFLD with the GEO2R online tool. Next, the Gene Ontology (GO) and Kyoto Encyclopedia of Genes and Genomes (KEGG) pathway enrichment analysis for the DEGs were performed. Then, the protein-protein interaction (PPI) network was constructed and the hub genes were identified using the STRING database and Cytoscape software. Finally, NetworkAnalyst was used to construct the network between the targeted microRNAs (miRNAs) and the hub genes.

**Results:** A total of 52 genes were identified as DEGs in the above two datasets. GO and KEGG enrichment analysis indicated that DEGs are mostly enriched in immunity and inflammation related pathways. In addition, nine hub genes, including TREM1, S100A9, FPR1, NCF2, FCER1G, CCR1, S100A12, MMP9, and IL1RN were selected from the PPI network by using the cytoHubba and MCODE plug-in. Then, four miRNAs, including miR-20a-5p, miR-129-2-3p, miR-124-3p, and miR-101-3p, were predicted as possibly the key miRNAs through the miRNA-gene network construction.

**Conclusion:** In summary, we firstly constructed a miRNA-gene regulatory network depicting interactions between the predicted miRNA and the hub genes in NAFLD and PCOS, which provides novel insights into the identification of potential biomarkers and valuable therapeutic leads for PCOS and NAFLD.

## Introduction

PCOS is a heterogeneous disorder characterized by hyperandrogenemia, ovulatory dysfunction, and it is a common reproductive and endocrine disorder that mainly occurs in puberty and childbearing age, affecting 6%–20% of women (Rotterdam, 2004; Azziz et al., 2009; Rocha et al., 2019). Furthermore, PCOS is associated with multiple metabolic disorders, including insulin resistance (IR), obesity, dyslipidemia, type 2 diabetes (T2DM), and cardiovascular disease (Goyal and Dawood, 2017). PCOS is the most common cause of anovulatory infertility, and its high incidence has brought huge health and economic costs to the family and society (Homburg and obstetrics, 2004).

NAFLD as one of the most prevalent chronic liver diseases globally is a type of metabolic-related liver disease closely related to IR and genetic susceptibility that includes non-alcoholic simple hepatic steatosis (NAFL), non-alcoholic steatosis Hepatitis (NASH), cirrhosis and hepatocellular carcinoma (HCC) (Diehl and Day, 2017). NAFLD is commonly recognized as a hepatic manifestation of the metabolic syndrome (MS), and it is frequently accompanied by T2DM, obesity, and dyslipidemia (Wattacheril, 2020). According to statistics, the global prevalence of NAFLD was estimated to be 25% (Younossi et al., 2016). Therefore, NAFLD has become a health problem and an economic burden in the world.

In 2005, Brown et al. first found a potential relationship between PCOS and NAFLD (Brown et al., 2005). To date, there are more and more that studies have substantiated a significant link between NAFLD and PCOS (Kelley et al., 2014). Some reports have suggested that 68 the prevalence of NAFLD in women with PCOS is significantly higher than that in 69 non-PCOS women (Falzarano et al., 2022). PCOS patients can promote the occurrence and development of NAFLD through various factors such as hyperandrogenism, IR, obesity, dyslipidemia, chronic low-grade inflammation, and intestinal flora imbalance (Vassilatou, 2014; Boursier et al., 2016; Kim et al., 2017; Yurtda ş and Akdevelioğlu, 2020). Although the relationship between NAFLD and PCOS has attracted substantial interest recently, leading to a vast amount of research related to this topic, the related genetics research is still limited and needs further exploration. There is a need for further studies to investigate NAFLD in the context of PCOS, as well as potential therapeutic options.

In recent years, microarray technology and bioinformatic analysis have been widely used to screen genetic alterations at the genome level. In the present study, we for the first time analyzed two original microarray datasets that were downloaded from Gene Expression Omnibus (GEO, http://www.ncbi.nlm.nih.gov/geo) to obtain DEGs between NAFLD and PCOS. Subsequently, the identified DEGs were analyzed by Gene Ontology (GO) analysis, Kyoto Encyclopedia of Genes and Genomes (KEGG) pathway analysis and protein-protein interaction (PPI) analysis. Then, we predicted the potential target miRNAs of DEGs, and constructed a visualized miRNA-gene interaction network through Cytoscape software (http://www.cytoscape.org/). The present study aimed to identify hub genes and hot research topic.

## Materials and Methods

### Gene Expression Profile Data Collection

Based on the GPL570 platform, two microarray datasets (GSE63067, GSE34526) of PCOS, NAFLD and control samples were collected from the GEO database. The **GSE34526** data set consisted of the gene expression profiles of seven PCOS patients and three normal controls. The **GSE63067** data set included 18 samples, of which 11 were NAFLD patients, and seven were controls.

### Differentially Expressed Genes Selection

DEGs were extracted and analyzed separately using the GEO2R online analysis tool (https://www.ncbi.nlm.nih.gov/geo/geo2r/), which is an R-based web application included in the GEO database. Adjusted *p* values were used to reduce the false-positive rate using Benjamini and Hochberg method. The threshold of DEGs screening was ∣log2 FC | ≥1 and *p* < 0.05. The DEGs obtained from the two datasets were visualized using the R packages “complexheatmap” and “ggplot2” to generate the heat maps and volcano maps, respectively. In addition, the overlapping DEGs between PCOS and NAFLD were delineated using the Venn diagrams with the Venn online platform (http://bioinformatics.psb.ugent.be/webtools/Venn/). These overlapping DEGs were retained for subsequent analysis.

### Functional Classification and Pathway Enrichment of DEGs

The above overlapping DEGs were submitted to GO function enrichment analysis, which consisted of biological process (BP), cellular component (CC), and molecular function (MF), and the KEGG signaling pathway enrichment analysis using an R package “clusterProfiler”. The enriched GO terms and KEGG pathways with an adjusted *p* value < 0.05 was selected.

### Protein-Protein Interaction Establishment and Identification of hub Genes

To further explore the interaction among the common genes obtained above, the Search Tool for the Retrieval of Interacting Genes (STRING) (http://string-db.org/) was used for PPI network construction. A minimum interaction score of above 0.4 was considered to be significant. Subsequently, Cytoscape software was used to visualize the PPI network. And then, we used the Cytoscape plug-in Minimal Common Oncology Data Elements (MCODE, http://apps.cytoscape.org/apps/mcode) to screen out key protein expression molecules. Then, the Maximal Clique Centrality (MCC) algorithm in the cytoHubba plug-in (http://hub.iis.sinica.edu.tw/cytohubba/) was applied to screen the hub genes with high connectivity in PPI networks.

### MiRNAs Associated With hub Genes

NetworkAnalyst tool (version 3.0, https://www.networkanalyst.ca/) was used to construct the miRNA-gene interactions of the hub genes. Finally, these hub genes and miRNA were plotted through Cytoscape software.

## Results

### Identification of DEGs in PCOS and NAFLD

**FIGURE 1:**
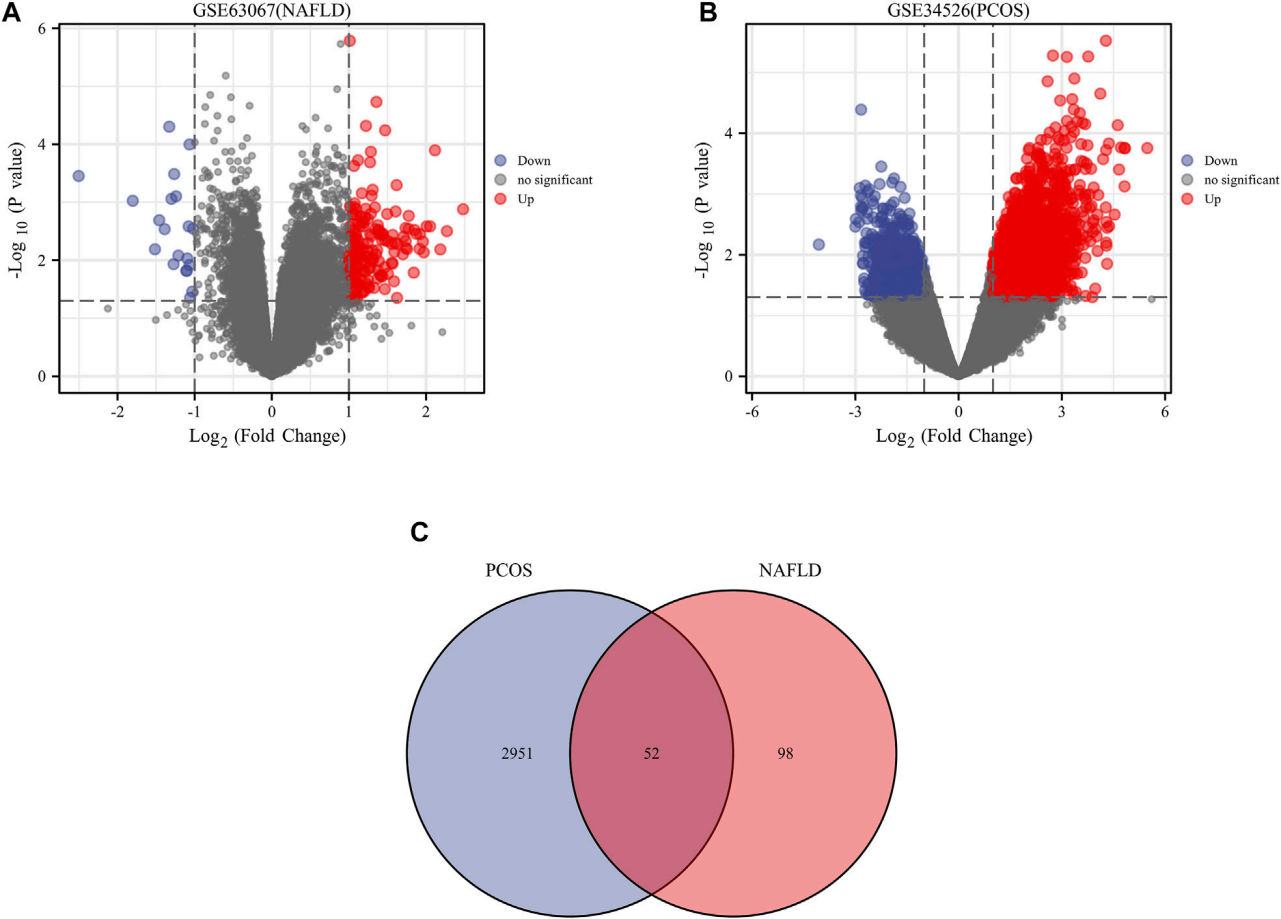
The volcano plot and venn diagram of differentially expressed genes (DEGs). Panel **(A)**, volcano plot of DEGs in GSE34526; Panel **(B)**, volcano plot of DEGs in GSE63067; Panel **(C)**, venn diagram of DEGs in GSE34526 and GSE63067 gene chips. There are also two vertical dashed lines in the figure, representing log2 FC at −1 and 1; The horizontal dashed line represents adjusted *p* value at 0.05. Abbreviations: DEGs, differentially expressed genes; PCOS, Polycystic ovary syndrome; NAFLD, non-alcoholic fatty liver.

**FIGURE 2:**
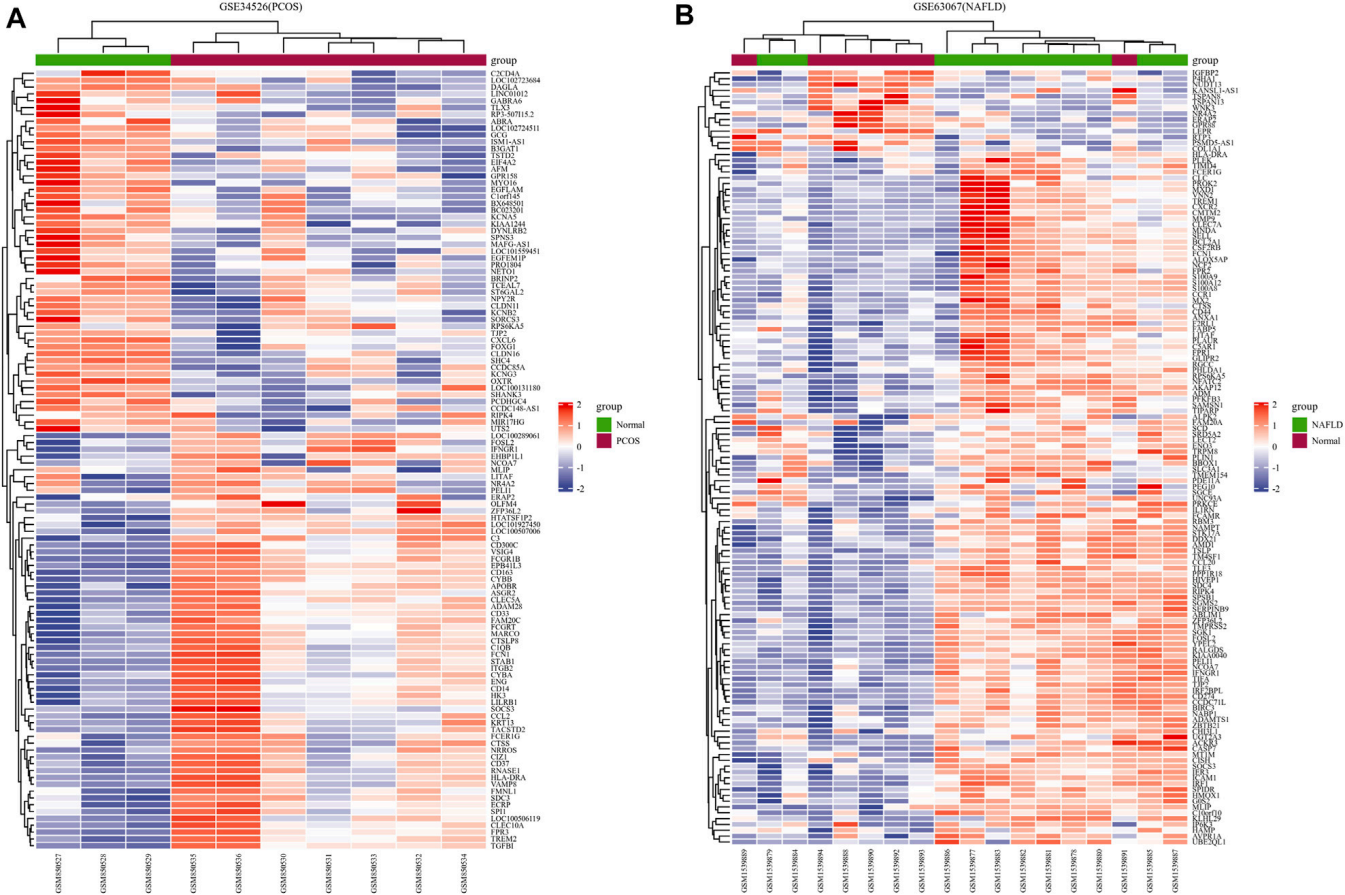
The heatmaps of differentially expressed genes (DEGs). Panel **(A)**, heatmap of DEGs in GSE34526; Panel **(B)**, heatmap of DEGs in GSE63067. Red represents upregulated DEGs, blue represents downregulated DEGs, and white represents no significant changes. Abbreviations: DEGs, differentially expressed genes; PCOS, Polycystic ovary syndrome; NAFLD, non-alcoholic fatty liver.

We downloaded the series GSE34526 dataset about PCOS and the series GSE63067 dataset about NAFLD from the NCBI GEO database. After screening with the threshold of an adjusted *p* value < 0.05 and ∣log2 FC | >1.0, 3003 DEGs (2176 upregulated and 827 downregulated) were identiﬁed in the GSE34526 dataset, 150 DEGs (133 upregulated and 17 downregulated) were identiﬁed in GSE63067 using the “limma” package in R software. The volcano plot and heatmap analyses were used to visualize the DEGs of the two data sets were shown in Figure 1 and Figure 2, respectively. In addition, a Venn diagram analysis was performed to evaluate the common DEGs between GSE34526 and GSE63067. As presented in Fig. 1C, 52 overlapping DEGs were identified.

### GO and KEGG Enrichment Pathway Analysis of Overlapping DEGs

**FIGURE 3:**
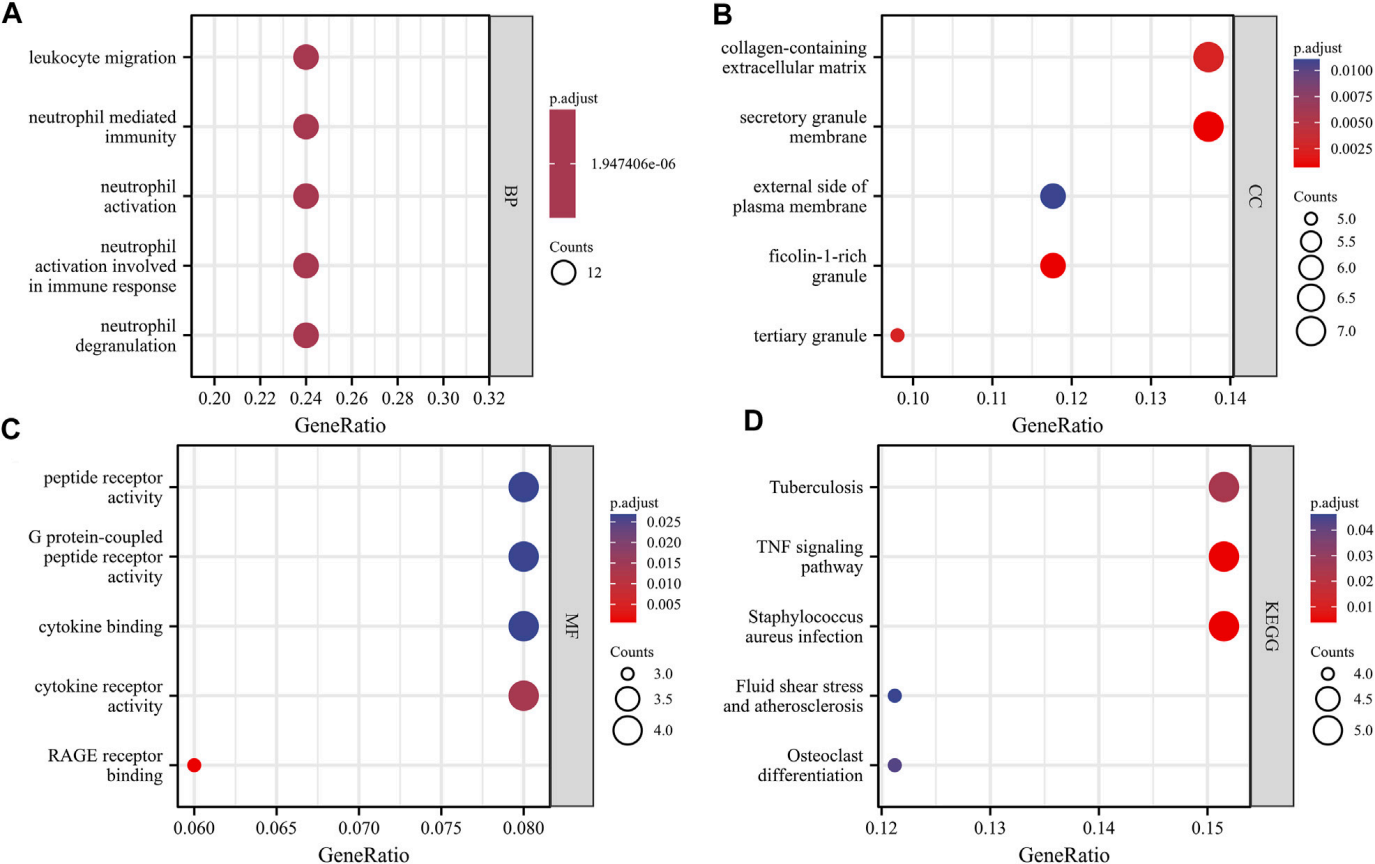
GO and KEGG analysis results of overlapping DEGs. Panel **(A)**, GO biological process enrichment results; Panel **(B)**, GO cell component enrichment results. Panel **(C**), GO molecular function enrichment results. Panel **(D)**, KEGG pathway enrichment results. An adjusted *p* < 0.05 was identified as significantly changed GOs and KEGG. The *x*-axis shows the gene ratio of each term. The *y*-axis represents the annotation items of GO-BP in A, the annotation of GO-CC in B, the annotation of GO-MF in C, and the annotation of KEGG in D. The bubble size represents the number of genes associated with each term. The color of each bubble represents the adjusted *p* value: the redder the color, the higher the enrichment. Abbreviations: GO, Gene Ontology; BP, biological process; CC, cellular component; MF, molecular function; KEGG, Kyoto Encyclopedia of Genes and Genomes.

With the clusterProfiler package in R software, we performed GO and KEGG pathway enrichment analyses to better understand the biological functions of the identified DEGs. After screening with the threshold of adjusted *p* < 0.05, we select the top five significantly enriched GO terms and top five KEGG terms. The results showed that DEGs were enriched in biological processes, including neutrophil degranulation, neutrophil activation involved in immune response, neutrophil activation, neutrophil mediated immunity, and leukocyte migration (Figure 3, Supplementary Table S1). In terms of cell component, DEGs were principally associated with ficolin-1-rich granule, secretory granule membrane, tertiary granule, collagen-containing extracellular matrix, and external side of the plasma membrane (Figure 3, Supplementary Table S1). The analysis of molecular function indicated that DEGs significantly enriched in RAGE receptor binding, cytokine receptor activity, cytokine binding, G protein-coupled peptide receptor activity, and peptide receptor activity (Figure 3, Supplementary Table S1). The top five significant KEGG pathways of DEGs were enriched in *Staphylococcus aureus* infection, tumor necrosis factor (TNF) signaling pathway, *Tuberculosis*, Osteoclast differentiation, and Fluid shear stress and atherosclerosis (Figure 3, Supplementary Table S1).

### PPI Network Construction and hub Genes Identification

**FIGURE 4:**
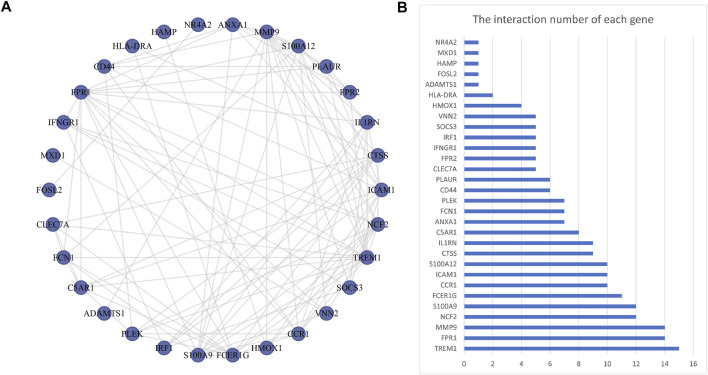
Protein-protein interaction network. Panel **(A)**, The PPI among overlapping DEGs. Panel **(B)**, The interaction number of each DEG. Abbreviations: DEGs, differentially expressed genes; PPI, protein-protein interaction.

**FIGURE 5:**
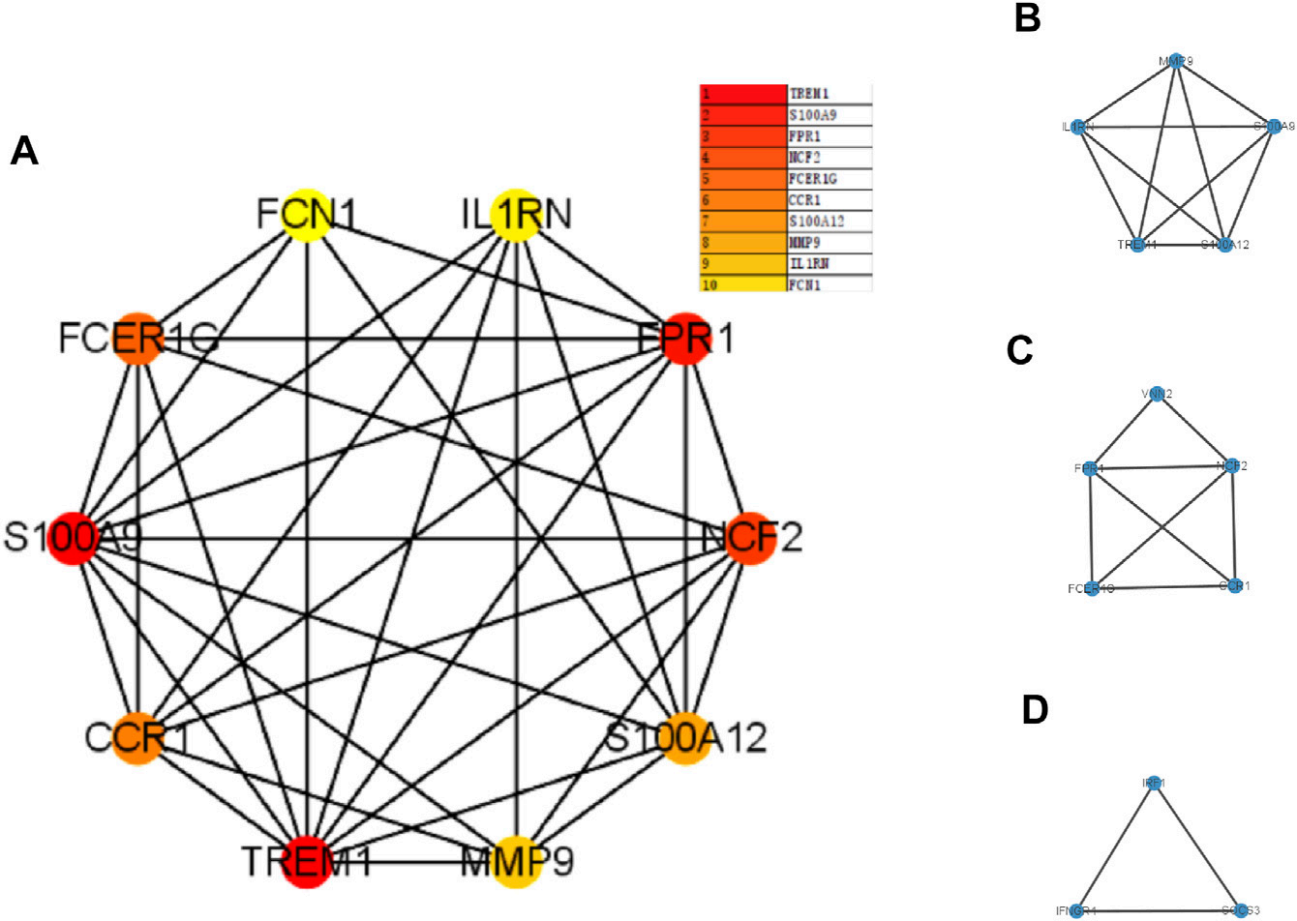
The PPI network analyzed by cytoHubba and MCODE. Panel **(A)**, PPI network for the top 10 hub genes. Panel **(B–D)**, three cluster modules extracted by MCODE. Abbreviations: PPI, protein-protein interaction; MCODE, Minimal Common Oncology Data Elements.

The PPI network was first performed based on the STRING database to determine the interactions among the overlapping DEGs. Then, the obtained results were imported into Cytoscape software for visual analysis (Figure 4A). And the interaction number of each gene was also shown (Figure 4B). The PPI network was analyzed by Cytoscape plug-in cytoHubba to identify hub genes. Based on the MCC algorithm, the top 10 genes were identified as potential hub genes: Triggering receptor expressed in myeloid cells 1 (TREM1), S100 calcium binding protein A9 (S100A9), Formyl peptide receptor 1 (FPR1), Neutrophil cytosolic factor 2 (NCF2), Fc fragment Of IgE receptor Ig (FCER1G), C-C chemokine receptor type 1 (CCR1), S100A12 (S100 calcium binding protein A12), Matrix metalloproteinases 9 (MMP9), and IL-1 receptor antagonist (IL1RN) (Figure 5A). Next, the MCODE plug-in was used to identify significant gene clusters modules and obtain cluster scores (filter criteria: degree cut-off = 2; node score cut-off = 0.2; k-core = 2; max depth = 100), and three modules were obtained, which were shown in Figures 5B–D. These modules contained 13 potential hub genes: IL1RN, MMP9, S100A9, TREM1, S100A12, NCF2, FPR1, FCER1G, CCR1, VNN2, SOCS3, IRF1, and IFNGR1. We took the intersection of the hub gene obtained by cytoHubba and the hub gene obtained by MCODE to obtain nine hub genes: TREM1, S100A9, FPR1, NCF2, FCER1G, CCR1, S100A12, MMP9, and IL1RN.

### Target miRNAs Prediction and Integrated miRNAs-Targets Network Construction

**FIGURE 6:**
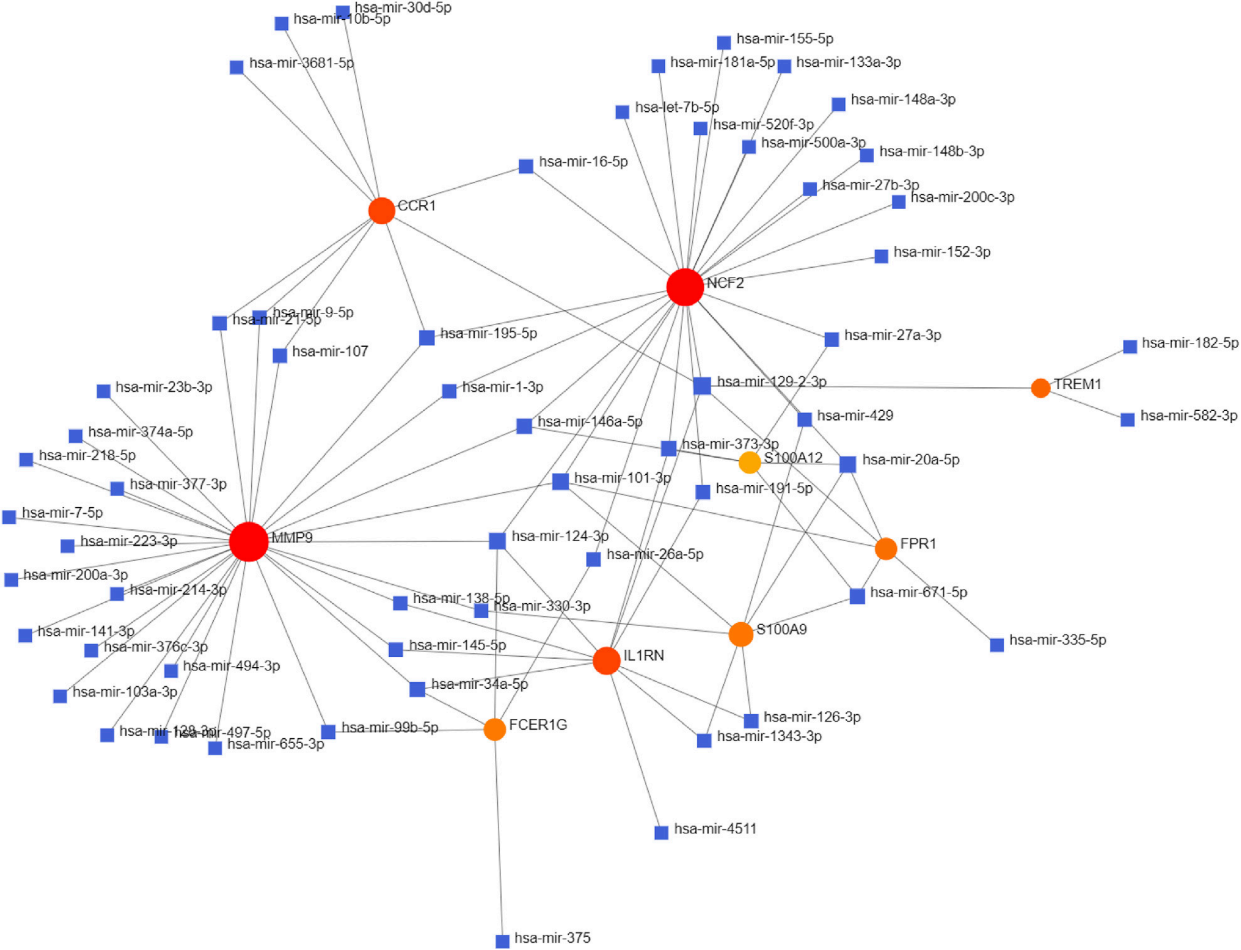
Integrated miRNA-gene interaction networks for the nine hub genes. Red circles represent nine hub genes. Blue squares represent miRNA which has connectivity with hub genes. Abbreviations: miRNA, microRNA.

The NetworkAnalyst databases were used to anticipate target miRNAs of hub genes. We used Cytoscape software to construct the miRNA-gene interaction network, which comprised 67 nodes and 96 edges. As shown in Figure 6, NCF2, S100A9, and FPR1 could be interacted with two common target miRNAs: miR-20a-5p and miR-101-3p. miR-129-2-3p was interacted with four hub genes, including TREM1, NCF2, IL1RN, CCR1, and FPR1. miR-124-3p was interacted with four hub genes, including NCF2, FCER1G, MMP9, and IL1RN. However, these findings need further validation.

## Discussion

Nowadays, more and more studies have confirmed the correlation between PCOS and NAFLD. A previous study showed that the prevalence of NAFLD was significantly higher in women with PCOS than in those without, with a prevalence of 51.56 and 29.64%, respectively (Falzarano et al., 2022). Meanwhile, a study revealed that PCOS was also more prevalent in female NAFLD patients with a prevalence of 50% up to a high of 70%, which is significantly higher than in those without NAFLD (Georgescu, 2022). Furthermore, studies have shown that both PCOS patients and NAFLD patients can increase the risk of cardiovascular disease (Dokras, 2019), and metabolic abnormalities were more pronounced in the PCOS patients with concurring NAFLD (Sarkar et al., 2020). In addition, cardiovascular disease is the most common cause of death in NAFLD patients (Söderberg et al., 2010). So far, the mechanism of the link between NAFLD and PCOS is not fully understood. Therefore, exploring the molecular mechanisms between PCOS and NAFLD, early identification and intervention, undoubtedly have important clinical significance.

In the present study, a series of bioinformatics analyses were performed on two independent gene chip databases of PCOS and NAFLD, and 52 common DEGs between PCOS and NAFLD were obtained based on the GEO database. The result of GO enrichment analysis indicated that the DEGs were mainly enriched in immune response, neutrophil activation, neutrophil mediated immunity, and leukocyte migration. This result suggests that the DEGs between PCOS and PCOS are related to inflammation and immune response, which is consistent with previous studies (Moulana, 2019; Oates et al., 2019). In addition, KEGG analysis showed that the DEGs were mainly enriched in *Staphylococcus aureus* infection, TNF signaling pathway, *Tuberculosis*, Osteoclast differentiation, and Fluid shear stress and atherosclerosis. We further classified these pathways according to the KEGG pathway Database. We found that they are mainly related to immunity and inflammation. Many studies have shown that immune inflammatory processes and atherosclerosis play a critical role in cardiovascular diseases (Zhang et al., 2020). Moreover, PCOS and NAFLD are strongly associated with cardiovascular diseases (Scicchitano et al., 2012; Niederseer et al., 2021). Our findings provide new ideas that patients with PCOS and NAFLD are more prone to cardiovascular disease.

Subsequently, according to the MCODE plug-in and cytoHubba plug-in of Cytoscape, we screened nine overlapping DEGs as hub genes in the PPI network, including TREM1, S100A9, FPR1, NCF2, FCER1G, CCR1, S100A12, MMP9, and IL1RN. These nine genes were all upregulated in both PCOS patients and NAFLD patients, suggesting these genes may play important role in the mechanism of PCOS and NAFLD.

S100A9 and S100A12 belong to a family of 25 homologous low‐molecular‐weight intracellular calcium‐binding proteins produced by cells of myeloid origin (Donato and biology, 2001). S100A9 is mainly released by activated granulocytes and associated with various types of inflammation-related pathways (Wang et al., 2018). A previous study showed that S100A9 could increase the production of inflammatory cytokines and disturb steroidogenesis *via* activating nuclear factor kappa B (NF‐κB) signaling pathway in PCOS (Li et al., 2020). Furthermore, S100A9 promotes inflammation and lipolysis in the liver during NAFLD progression (Wu et al., 2020). Another study demonstrated that S100A9 can differentiate hepatic and metabolic progression in NAFLD as a biomarker in NAFLD progression (Liu et al., 2015). Additionally, studies have shown that S100A12 interacts with S100A9 (Hatakeyama et al., 2004) and also has proinflammatory activity (Miranda et al., 2001). There are no reports of S100A12 in PCOS and NAFLD. We speculate that S100A9 and S100A12 synergistically promote inflammation in PCOS and NAFLD. Interestingly, S100A12 has also been shown to block the activity of S100A9 (Katte and Yu, 2018).

NCF2 is a rate-limiting cofactor of NADPH oxidase that is necessary for reactive oxygen species (ROS) production in phagocytes, which plays a critical role in innate immunity and phagocytic microbicidal activity (Muise et al., 2012). NCF2 is highly expressed in PCOS and NAFLD and contributes to oxidative stress in PCOS and NAFLD (Kaur et al., 2012; Garcia-Jaramillo et al., 2019).

IL1RN is originally discovered as a natural antagonist of IL-1 (Arend, 1991), which encodes the IL1 antagonist protein (IL1RA) (Gabay et al., 2010). And IL1RA has been reported as a critical mediator of inflammatory processes and plays a vital role in the pathogenesis of PCOS (Xia et al., 2013). IL1RN also plays an important role in the development of NAFLD (Wolfs et al., 2015).

MMP9 is one of the zinc-ion-dependent metalloproteinase family, playing a pathogenic role in chronic inflammatory autoimmune diseases (Akter et al., 2015). MMP9 was shown to be overexpressed in PCOS and involved in the pathogenesis of PCOS (Ranjbaran et al., 2016). MMP9 could increase fibrosis severity in NAFLD and be involved in the development and progression of human HCC metastasis (Shi et al., 2010; Goyale et al., 2021). TREM1 is expressed on myeloid lineage cells and played an important role in maintaining homeostasis and the normal immune responses. Dou et al. demonstrated that TREM1 in the liver was involved in the pathological process and therapeutic outcome of NASH (Dou et al., 2016). In addition, TREM1 promotes atherosclerosis by inducing monocytosis and foam cell formation (Zysset et al., 2016). FPR1 is a member of 7-transmembrane G protein-coupled receptors (GPCRs), which is mostly expressed by myeloid cells and is a key regulator of the inflammatory environment (Dahlgren et al., 2016; Mastromarino et al., 2019). FCER1G is engaged in a variety of immune responses as a constitutive component of the high-affinity immunoglobulin E (Ig E) receptor and interleukin-3 receptor complex (Liang et al., 2013). CCR1 is a member of the seven-transmembrane G-protein-coupled receptor family, which is widely expressed on myeloid cells and is involved in the activation and trafficking of immune cells (Schaller et al., 2008). Based on previous research, these nine hub genes have been shown to have key effects on immune response and inflammatory response. Moreover, it has been known that immune dysregulation and chronic inflammation are important factors in the development of PCOS and NAFLD (Anstee et al., 2013; Qin et al., 2016). Based on this, we speculate that: these genes may also contribute to the occurrence and development of PCOS and NAFLD, and may be potentially new treatment targets in the future.

MiRNAs are small non-coding RNA molecules (21–25 nucleotides long) that are partially or fully complementary to the 3′ URT of the target gene mRNA by inducing mRNA degradation or repressing mRNA translation (He and Hannon, 2004). In this study, we also constructed a miRNA-target gene network and selected the four miRNAs (miR-20a-5p, miR-129-2-3p, miR-124-3p, and miR-101-3p) that interacted the most with DEGs. miR-20a-5p was found to bind to pro-angiogenic genes and suppress their expression and thus exerting anti-angiogenic activity in PCOS (Patil et al., 2022). miR-20a-5p also played a protective role in lipid metabolism and IR of NAFLD (Wang et al., 2020; Zhang et al., 2021). Previous studies on miR‐129-2-3p mainly focused on cancer. miR-129-2-3p has not been reported in PCOS and NAFLD. A study has shown that miR-129-2-3p was involved in the functional regulation of neutrophils in chronic inflammatory processes. Thus, we speculate that miR-129-2-3 may be involved in the pathophysiology of PCOS through inflammatory processes. miR-124-3p was downregulated in PCOS patients (Ding et al., 2015) and was involved in lipid metabolism in NAFLD as a critical regulator in lipid homeostasis of the liver (Wang et al., 2020918). miR-101-3p was also downregulated in PCOS patients (Butler et al., 2020). Moreover, the downregulation of miR-101-3p was more strongly associated with hepatic injury in NAFLD (Meroni et al., 2019).

In conclusion, our study identified some hub genes and speculated that miRNA-gene regulatory networks contribute to the pathophysiological process of PCOS and NAFLD by bioinformatics analysis, which provided the potential diagnostic and therapeutic targets of PCOS and NAFLD. However, there are some limitations to our study. Firstly, only the top nine hub genes were involved in our current study. Secondly, a lack of research on detailed molecular mechanisms that the hub genes and miRNAs regulate in PCOS and NAFLD. Thirdly, the miRNA-gene interaction networks were only based on predictions from public databases. Thus, the molecular mechanism of these hub genes and miRNAs in the occurrence of PCOS and NAFLD need to be further studied.

## Conclusion

Our study identified a miRNA-gene network potentially relevant for PCOS and NAFLD. The nine hub genes (including TREM1, S100A9, FPR1, NCF2, FCER1G, CCR1, S100A12, MMP9, and IL1RN) were significantly upregulated, which may have a critical influence on the pathophysiological mechanism of PCOS and NAFLD. Some potential target miRNAs (miR-20a-5p, miR-129-2-3p, miR-124-3p, and miR-101-3p) were also predicted and may participate in the pathophysiological process of PCOS and NAFLD through inflammation and immune response. These findings may contribute to the development of early diagnostic strategies, prognostic markers, and therapeutic targets.

## Data Availability

The datasets presented in this study can be found in online repositories. The names of the repository/repositories and accession number(s) can be found in the article/Supplementary Material.
