# Supplementary material for: Key Genes Associated With Non-Alcoholic Fatty Liver Disease and Polycystic Ovary Syndrome
Source: Front Mol Biosci. 2022 May 25;9:888194. doi: 10.3389/fmolb.2022.888194 (PMC9174783; doi:10.3389/fmolb.2022.888194)
Supplement: Supplementary file 1 [file Table1.DOCX]

| Ontology | ID | Description | GeneRatio | BgRatio | pvalue | p.adjust | qvalue |
| --- | --- | --- | --- | --- | --- | --- | --- |
| BP | GO:0043312 | neutrophil degranulation | 12/50 | 485/18670 | 4.09e-09 | 1.95e-06 | 1.28e-06 |
| BP | GO:0002283 | neutrophil activation involved in immune response | 12/50 | 488/18670 | 4.38e-09 | 1.95e-06 | 1.28e-06 |
| BP | GO:0042119 | neutrophil activation | 12/50 | 498/18670 | 5.49e-09 | 1.95e-06 | 1.28e-06 |
| BP | GO:0002446 | neutrophil mediated immunity | 12/50 | 499/18670 | 5.62e-09 | 1.95e-06 | 1.28e-06 |
| BP | GO:0050900 | leukocyte migration | 12/50 | 499/18670 | 5.62e-09 | 1.95e-06 | 1.28e-06 |
| CC | GO:0101002 | ficolin-1-rich granule | 6/51 | 185/19717 | 7.98e-06 | 6.93e-04 | 5.56e-04 |
| CC | GO:0030667 | secretory granule membrane | 7/51 | 298/19717 | 1.10e-05 | 6.93e-04 | 5.56e-04 |
| CC | GO:0070820 | tertiary granule | 5/51 | 164/19717 | 6.46e-05 | 0.002 | 0.002 |
| CC | GO:0062023 | collagen-containing extracellular matrix | 7/51 | 406/19717 | 7.90e-05 | 0.002 | 0.002 |
| CC | GO:0009897 | external side of plasma membrane | 6/51 | 393/19717 | 5.10e-04 | 0.011 | 0.009 |
| MF | GO:0050786 | RAGE receptor binding | 3/50 | 11/17697 | 3.45e-06 | 6.20e-04 | 4.61e-04 |
| MF | GO:0004896 | cytokine receptor activity | 4/50 | 96/17697 | 1.55e-04 | 0.014 | 0.010 |
| MF | GO:0019955 | cytokine binding | 4/50 | 128/17697 | 4.65e-04 | 0.027 | 0.020 |
| MF | GO:0008528 | G protein-coupled peptide receptor activity | 4/50 | 146/17697 | 7.63e-04 | 0.027 | 0.020 |
| MF | GO:0001653 | peptide receptor activity | 4/50 | 152/17697 | 8.86e-04 | 0.027 | 0.020 |
| KEGG | hsa05150 | Staphylococcus aureus infection | 5/33 | 96/8076 | 3.90e-05 | 0.004 | 0.003 |
| KEGG | hsa04668 | TNF signaling pathway | 5/33 | 112/8076 | 8.17e-05 | 0.004 | 0.003 |
| KEGG | hsa05152 | Tuberculosis | 5/33 | 180/8076 | 7.44e-04 | 0.025 | 0.020 |
| KEGG | hsa04380 | Osteoclast differentiation | 4/33 | 128/8076 | 0.002 | 0.043 | 0.035 |
| KEGG | hsa05418 | Fluid shear stress and atherosclerosis | 4/33 | 139/8076 | 0.002 | 0.046 | 0.037 |

Supplementary Table 1

Results of GO and KEGG analysis
